# Nanotechnology and Reproductive Management of Farm Animals: Challenges and Advances

**DOI:** 10.3390/ani11071932

**Published:** 2021-06-29

**Authors:** Nesrein M. Hashem, Antonio Gonzalez-Bulnes

**Affiliations:** 1 Department of Animal and Fish Production, Faculty of Agriculture (El-Shatby), Alexandria University, Alexandria 21545, Egypt; 2 Departamento de Produccion y Sanidad Animal, Facultad de Veterinaria, Universidad CardenalHerrera-CEU, CEU Universities, C/Tirant lo Blanc, 7, 46115 Alfara del Patriarca, Valencia, Spain

**Keywords:** nano-delivery system, reproductive management, bio stimulation, nutrition, hormones, antibiotics, reproductive diseases, livestock

## Abstract

**Simple Summary:**

The emergence of nanotechnology paves the way for innovating countless applications in the agricultural and livestock production sector. In the field of reproductive management of farm animals, nanotechnology offers unconventional and innovative solutions for the existing reproductive management challenges. The main concept of nanotechnology comes through their ability to modulate drug behavior and consequently their biological effects (e.g., male effect). In this review, the challenges of the current reproductive management in farm animals will be discussed in line with the possible solutions offered by applying nanotechnology.

**Abstract:**

Reproductive efficiency of farm animals has central consequences on productivity and profitability of livestock farming systems. Optimal reproductive management is based on applying different strategies, including biological, hormonal, nutritional strategies, as well as reproductive disease control. These strategies should not only guarantee sufficient reproductive outcomes but should also comply with practical and ethical aspects. For example, the efficiency of the biological- and hormonal-based reproductive strategies is mainly related to several biological factors and physiological status of animals, and of nutritional strategies, additional factors, such as digestion and absorption, can contribute. In addition, the management of reproductive-related diseases is challenged by the concerns regarding the intensive use of antibiotics and the development of antimicrobial resistant strains. The emergence of nanotechnology applications in livestock farming systems may present innovative and new solutions for overcoming reproductive management challenges. Many drugs (hormones and antibiotics), biological molecules, and nutrients can acquire novel physicochemical properties using nanotechnology; the main ones are improved bioavailability, higher cellular uptake, controlled sustained release, and lower toxicity compared with ordinary forms. In this review, we illustrate advances in the most common reproductive management strategies by applying nanotechnology, considering the current challenges of each strategy.

## 1. Introduction

In livestock farming systems, reproductive efficiency has central consequences on the productivity, profitability, and sustainability of farms. The reproductive performance of farm animals determines the efficiency of milk and/or meat production, either directly or through managing decisions, such as replacement and culling rates. Optimal reproductive management is based on applying precision strategies which also needs to consider costs, animals’ welfare, environmental impacts, and human health. Most of the reproductive management practices are ready for their use in commercial livestock farms after selecting the strategy which meets goals of every farm [1,2]. Such strategies may include one or more bio stimulation tools (e.g., male effect), reproductive assisted techniques (mainly estrous synchronization and artificial insemination), nutritional management, and prevention/treatment of reproductive diseases [3,4,5].

Although these reproductive management strategies are widely and predominantly applied in different livestock production systems, their efficiency is challenged by several practical and ethical aspects. For example, hormonal-based reproductive therapies are the preferred method for reproductive management; however, their effectiveness is highly dependent on their pharmacokinetics and pharmacodynamics, which may be affected by biological factors [6,7]. The male effect is a sexual bio stimulation method that confers an opportunity to eliminate the intensive use of hormones in reproductive management and the production of hormone residues-free animal products; however, its outputs are challenged by the sexual activity of both males and females, male to female ratio, and age and the experience of the male [4,8,9,10]. Similarly, nutritional management practices for improving the reproductive performance of farm animals may be negatively impacted by the lack of nutrients bioavailability and insufficient delivery of required nutrients [11,12]. Lastly, the management of reproductive-related diseases is challenged by the concerns regarding the intensive use of antibiotics and the development of antimicrobial-resistant strains [13,14,15].

In view of these considerations, the emergence of novel technologies, such as nanotechnology, paves the way for countless applications in agricultural and livestock production. The most important and promising application of nanotechnologies in the livestock production sector is in the field of nano-drug delivery systems. Many drugs, biological molecules, and nutrients can acquire novel physicochemical properties by using nanotechnology, such as improved bioavailability, higher mobility and cellular uptake, controlled sustained release of the drug at the target site, lower toxicity compared with other compounds, improved enzymatic actions, and increased mucoadhesive properties [16]. In this review, we aim to illustrate possible advances in the most common reproductive management strategies by applying nanotechnology.

## 2. Biological Stimulation Management, Male Effect

### 2.1. Challenges of Male Effect Applications

Pheromones are volatile chemical substances secreted by specific organs and scent glands of different animals. In mammals, including livestock species, pheromones play crucial roles in the communication between animals, signaling many behavioral and physiological processes. The role of pheromones in regulating several reproductive events in farm animals is well documented and can be indicated through observing changes in animals’ activity following direct (physical) and/or indirect (spatial) exposure to specific sexual pheromones [4,17]. The most known pheromones-based phenomenon that can prime and regulate many reproductive events in farm animals is known as the “Male Effect” [18]. Pheromones in the wool, wax, and urine originating from sexually active males have a significant influence on the onset of puberty, the resumption of sexual activity of seasonal anestrous females, and the reduction in postpartum anestrus in many farm animals, specifically sheep, goats, and swine [19,20]. Each of these effects is mediated by olfactory chemical signals, pheromones, released from the male and affecting the hypothalamic system of the stimulated female to generate pulses of gonadotropin-releasing hormone (GnRH) modulating sexual activity [21]. The male effect is well-recognized and applied in reproductive management [22,23,24]. In small ruminants, for example, many breeds exert different degrees of seasonality, which constrains the progression of the breeding program of commercial flocks, being restricted within a definite period of the year [25]. The resumption of sexual and ovulation activities of the seasonal anestrous females depends on the activation of the neuro-endocrine system by male pheromone signals and the subsequent modulation in LH pulse frequency and amplitude after sudden exposure to males. Usually, ovulation occurs within 2–3 days after male introduction to a large number of females [26]. From a practical point of view, the male effect not only has the advantage of advancing the breeding season by about 4–6 weeks or more, but it can also provide an acceptable degree of female synchrony at the time of breeding and thus the subsequent lambing/kidding time. In context, the male effect is successfully used to induce ovulation in prepubertal ewe lambs and lactational anestrous ewes. The male effect can be used with 35 to 40 days postpartum ewes to decrease the time between deliveries [19].

In pigs, the presence of boars reduces the postpartum period in lactating sows and the age of onset of puberty in gilts by about 30 days. These effects were early linked toolfactory cues because a pen previously occupied by a boar and presumably pervaded with a boar’s odor is effective in inducing early puberty [17]. Apparently, priming pheromones remaining in the boar’s pen after his removal are sufficient to induce early puberty [21]. The same was observed for sheep and goats, as pheromones in the wool, wax, and urine are sufficient to stimulate females to ovulate.

The male effect, being a sexual bio stimulation method, confers an opportunity to diminish, or even eliminate in the future, the intensive use of hormones in reproductive management and promote the production of hormone residues-free animal products. However, the outputs of this method, when applied at a farm scale, are challenged by several limiting factors. In brief, the main such factors are latitude, breed, sexual activity, hormonal status, age, and experience of the male [8,9,10], the depth of female anestrous status [4], male to female ratio, and the length of isolation period [27]. In fact, the application of such a method for induction of estrus needs the isolation of males from females for a certain period and reintroduction thereafter to stimulate females’ sexual activity (estrus and ovulation) and/or to introduce new males that have not previously been kept in the herd [27]. In many cases, the male effect is applied during out of season, during which males also may undergo weak sexual activity. For this reason, many studies have referred to the importance of priming males with exogenous stimuli, such as hormonal treatments (mainly melatonin [28] and testosterone [8]) and photostimuli [29,30], to speed up the sexual activity of males during the natural anestrous season. Overall, these challenges can negatively impact the application of the male effect at a large/field scale as a biological reproductive management protocol [4].

### 2.2. Nanotechnology Approaches for Developing Male Effect Procedure

The male effect, as previously described, depends on the effect of olfactory signals generated by male pheromones on the hypothalamic–pituitary–gonadal axis of the females. Previous research has shown that the physical or visual contact between male and female is not necessary to affect the sexual behavior of the female. Early studies confirmed the capacity of priming pheromones collected from the wool, wax, and urine of rams to stimulate ewes to ovulate early in the breeding season [31,32]. Similarly, a jar containing the odor of the buck around the location of females can be used as an aid to detect estrus in goats due to the strong characteristic seasonal odor of bucks pheromones [21]. A recent study tested the effectiveness of a novel 3-molecule boar pheromone spray to improve the reproductive performance of sows (BOARBETTER) [33]. Meta-analysis of the results showed significant increases in total born piglets by 0.49 per litter and born alive piglets by 0.37 per litter, with the treatment being modulated by the age of the sow, sincethe treatment increased total born piglets by 0.88 (*p* < 0.05) per litter and pigs born alive by 0.73 (*p* < 0.05) pigs per litter when considering first to third parties. The overall conclusion of this study is that the management of animals’ olfactory environment could be a cost effective, safe, and meaningful reproductive management tool for improving reproductive performance of sows [33].

Hence, the delivery of sexual pheromones to females seems the main mechanism mediating the male effect. Actually, the initial olfactory perception starts with the binding of the volatile molecules of the pheromones with olfactory binding proteins in olfactory-specific cells, such as the main olfactory epithelium [18]. Thereafter, pheromone signals can be transferred through the airways of nasal mucosa to olfactory centers in the brain to stimulate the hypothalamic–pituitary region to release reproductive hormones secretion. Furthermore, pheromones can be transferred through systematic circulations after passing tight junctions of olfactory epithelial cells [18,34,35]. In this context, it was suggested that volatile molecules of the pheromones, in spray form, could be more effective than the liquid form of the pheromones for delivering signals through the main olfactory epithelium receptors [33].

An aerosol nano-drug delivery system may facilitate the delivery of different drugs, including pheromones, through the olfactory pathways, nose-to-brain delivery and/or through the systematic circulation into target sites, passing the brain–blood and other biological barriers [14,34]. Despite few studies performed on applications of an aerosol nano-drug delivery system in the field of livestock production and veterinary medicine, Pamungkas et al. [35] proved the efficiency of chitosan-TPP nanoparticles for nasal human chorionic gonadotropins (hCG) delivery to induce ovulation in cows. Moreover, many vaccines have been developed using nanomaterials to be delivered through the nasal pathway [14].

**Figure 1:**
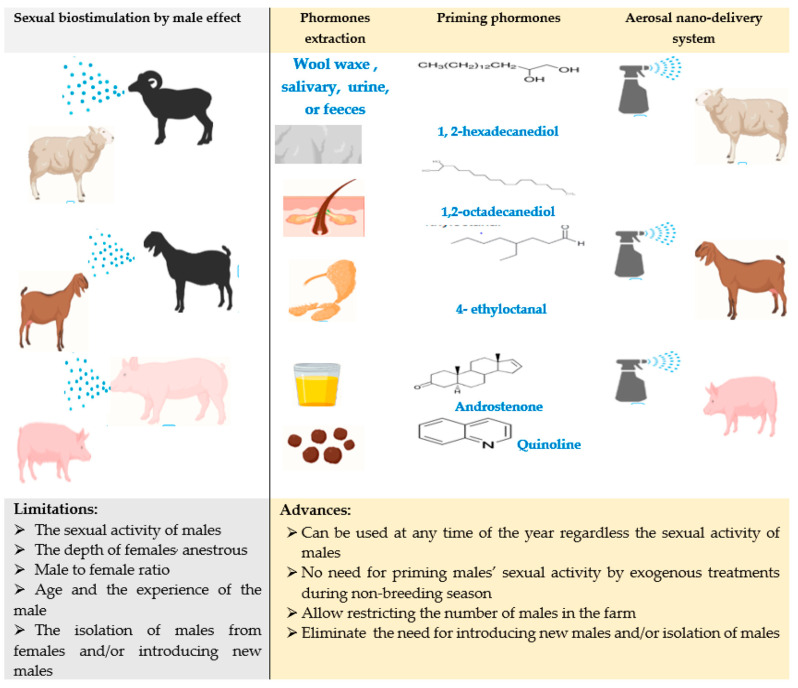
Possible applications of an aerosol nano-delivery system in pheromones-dependent sexual bio stimulation (male effect).

In this regard, different nanomaterials can be used as a novel delivery system to implement sexual priming pheromones ready for female’s treatment through nasal spray treatments. Specifically, the chemical compounds that act as pheromones are identified and can be isolated from different sites of the animal body including, wool wax, saliva, urine, and feces, of sexually active males. Fabrication of pheromones in an aerosol nano-formula may aid in overcoming some obstacles facing the male effect application at a farm scale. In this way, such therapies can be used at any season of the year regardless of the sexual activity of the males and the need for isolating or priming males’ sexual activitybefore introduction into the group of females. Moreover, it may also reduce the number of males on the farm (Figure 1). Actually, the emergence of aerosol nano-drug delivery technology opens the way for developing numerous pheromones-based protocols for biological control of reproductive events, such as the male effect, estrous detection, postpartum anestrous, puberty, pregnancy diagnosis, sexual activity of males, and mother-offspring relationship [36,37]. These could be achieved through producing commercial bio stimulating pheromones and/or pheromones-based bioassay kits. As an example, urinary pheromones 4-methyl phenol (4-mp, p-Cresol), 9-octadecenoic acid (oleic acid), and luteinizing hormone (LH) are suggested to develop nanoparticle-based bioassay kits for detection of estrus or ovulation in buffaloes [37].

## 3. Hormonal Based-Treatments

### 3.1. Importance and Challenges of Hormonal Based-Treatments

Currently, despite the attempts to use biological strategies for managing reproduction in farm animals, the procedures, including exogenous hormonal therapies, cannot be excluded from the farming system. Hormones-based treatments are an efficient tool to improve fertility and farm business profitability. Several survey studies confirmed the importance of hormonal-based protocols for reproductive management; e.g., around 80% of practitioners from 714 inorganic-dairy farms in England confirmed the importance of hormones for controlled and efficient reproductive management [38]; similarly, 87% (103 of 153) of managers from large dairy herds (average herd size of 613 cows) in the USA confirmed the use of hormonal synchronization or timed artificial insemination in their reproductive management programs [3]. Hormones are needed, and therefore widely used, for implementing different assisted reproductive techniques (estrous synchronization/induction, timed artificial insemination, and superovulation), improving reproductive efficiency, and treating reproductive pathologies in both females and males [8,39,40,41,42].

The most important hormones used for managing fertility in farm animals are gonadotrophins, progesterone, estradiol, testosterone, melatonin, and prostaglandins. The effectiveness of these hormones mainly depends on their pharmacokinetics and pharmacodynamics [6]. Some hormones, such as gonadotropin-releasing hormone (GnRH) and prostaglandin F_2α_ (PGF_2α_), have low molecular weights and a short lifespan, which restricts the sustained delivery of the hormones to the target sites and therefore their biological activity. Other hormones, such as glycoprotein gonadotropins (follicle-stimulating hormones, FSH; luteinizing hormone, LH; human chorionic gonadotropin, hCG; and equine chorionic gonadotropin, eCG), may stimulate the immune system and formation of specific antibodies, leading to a refractoriness to repeated gonadotropic treatments in different farm animal species [43,44]. In addition, repeated treatments with these hormones are associated with low fertility and other reproductive outcomes. It has been shown that anti-eCG antibodies can hindereCGbioactivitiesvia two mechanisms. First, through preventing the interaction of eCG with its receptors; second, by a conformational change in eCG by anti-eCG antibodies, which can inhibit eCG bioactivities. It is worth noting that these modulations of eCG bioactivities by its antibodiesmainly impact the FSH bioactivity of eCG rather than the LH bioactivity, which is essential for the recruitment and development of ovarian follicles, and therefore fertility is affected after repeated treatments [45,46]. In this context, ewes subjected up to three times to eCG/FSH based-super ovulatory treatments showed a lower fertilization rate and lower number of total recovered and viable embryos at the second and third recoveries when compared to the first flushing [44]. In another study, goats developing high eCG antibody concentrations after repeated eCG treatments exhibited a lower kiddingrate (41.3%) than other females (66.7%). The decreased fertility of these goats was associated with a delay in estrous rate and the preovulatory LH-surge [47]. Similarly, in rabbit does, repeated treatments with recombinant human FSH, increased FSH antibody levels in these females at the moment of the third and fourth superovulation treatments [43].

In addition to the aforementioned challenges facing the use of hormones for managing reproduction/fertility in farm animals, animal health and welfare and environmental-related issues are also raising challenges. For example, a shortage ofeCG availability is expected due to aspects related to animal welfare, as this hormone is obtained by bleeding pregnant mares. The ongoing societal pressure against companies manufacturing the hormone may prevent further hormone production in the near future [48].

Finally, conventional hormonal delivery systems may also upset the environmental ecosystem balance due to the spread of hormonal residues and carrier materials into the environment. The most evident example is the use of progesterone-impregnated intravaginal devices, which were developed for controlling the estrous cycle in different farm animals [49]. These devices are mainly composed of silicon polymers loaded with progesterone, which need to be loaded with high progesteroneconcentrations to release enough hormones to the vaginal mucosa, which increases the risk of hormone emissions to the environment and the transmission of the hormone directly to breeders/workers or indirectly to consumers through animal products [50].

### 3.2. Nanotechnology Approaches for Developing Hormonal Based-Treatments

Nano-delivery of hormones has emerged as a new pharmacological approach. Several engineered nanoparticles have been proposed as novel platforms for the protection and controlled release of reproductive hormones, including gonadotropin and steroid hormones. There are, to date, several studies reporting the use of nano-hormone delivery systems in the field of livestock reproductive management. These studies showed an enhancement of the pharmacokinetics and pharmacodynamics of the hormonal treatments, specifically, those of low molecular weight and short lifespan, such as GnRH [7,51,52,53,54]. In addition, the opportunity to use biodegradable materials as a matrix for progesteronedelivery instead of silicon-based materials was shown [55,56,57] to sustain the release of some hormones, such as melatonin in in vitro production media [58,59,60], and to change the route of hormone administration by enhancing mucosal absorption even if these hormones have high molecular weight [35]. Overall, these studies show that the use of nano-hormone delivery systems provides many advantages to hormonal-based treatments, such as decreasing hormone dosage, changing the route of administration, increasing animal welfare, and decreasing the risk of exposition to different hormones by workers and technicians (Table 1).

**Figure 2:**
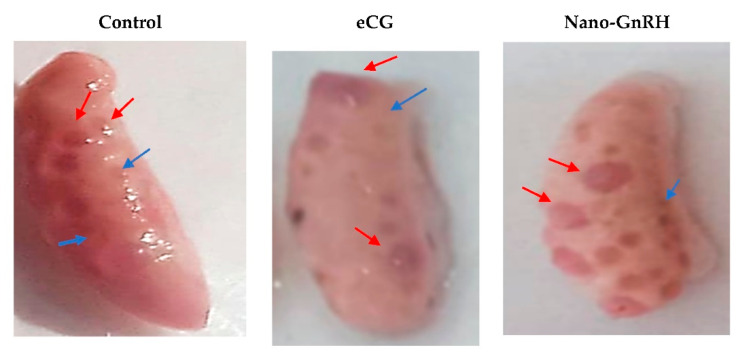
Two-days ovulation points (red arrows) and growing follicles (blue arrows) in ovaries of control rabbits or those treated with 25 IU equine chorionic gonadotropin (eCG) or 0.2µ gonadotropin-releasing hormone (GnRH)-chitosan- tripolyphosphate (TPP) nanoparticles. Higher numbers of growing follicles and ovulation points and larger diameters of ovulation points could be observed in GnRH-chitosan-TPP nanoparticles (unpublished data).

The more interesting trend is to use nano-hormone delivery systems to change the biological behavior of the hormones. This hypothesis is specifically raised in response to the direction towards the banning of eCG production and the lack of other products with similar activity. Among the different gonadotropins used for ovarian stimulation, eCG has a unique feature as it displays pronounced FSH-like activity in addition to its LH-like activity when used in species other than the equine [62,63]. Thus, the shortage/disappearance of this hormone will negatively impact many assisted reproductive techniques, specifically those depending on the stimulation of follicular growth. In these terms, Santos-Jimenez et al. [64] showed the possibility of the use GnRH as an eCG alternative if GnRH release behavior is slowed and sustained for a longer time, avoiding the occurrence of early LH-surge. In this study, diluting GnRH in propylene-glycol was effective in achieving this purpose and has been used as an eCG alternative in a CIDR-based estrous synchronization protocol tailored for sheep. Accordingly, nano-hormone delivery systems may aid in changing the biological behavior of GnRH. In these terms, a preliminary study by Hashem and co-authors (unpublished data), aimed at GnRH-chitosan-TPP nanoparticles as a potential alternative to eCG for stimulating ovarian follicles growth before artificial insemination in rabbits has been carried out. The results of this study showed the ability of GnRH-chitosan-TPP nanoparticles to induce greater follicular growth and formation of ovulation points than eCG following mating when it was subcutaneously administered at 2 µg/doe (Figure 2).

## 4. Nutritional Management

### 4.1. Importance and Challenges of Nutritional Management

Nutritional management is one of the most important strategies that can be used to control reproduction in different farm animals. Nutrition can affect the reproductive efficiency of farm animals at different reproductive windows, including the development of the reproductive organs during fetal life, puberty, and active breeding life [65]. Accurate synchronization between the periods of feeding and specific reproductive events enables achieving maximum benefits of nutrition. For example, feeding small ruminants with high energy-containing diets (concentrate and/or high energy feed additives, e.g., protected fats/glycerol) before breeding/mating season for a long term (about two months, [20,66]) or short term ( around 7–10 days before mating, [12]) has been found to improve reproductive performance of both males (spermatogenesis, semen quality, and libido; [24]) and females (folliculogenesis, ovulation rate, and estrous rate; [12]), leading to improved fecundity. In dairy animals, specifically in high milk-producing animals, proper nutritional management of the transition period (late pregnancy to early lactation) aids in decreasing the risks of negative energy balance and its consequences on animal health and postpartum reproductive performance (resumption of ovarian cycles and estrus and embryo survival) [67,68].

It is worth mentioning that the relationship between nutrition and reproduction is far more than meeting energy and protein requirements or maintaining body weight and body condition score [4]. The role of specific nutrients in controlling specific reproductive events is also described in enormous studies and has been known as “functional effects” [68,69,70]. In this regard, amino acids have been shown as one of the most functional nutrients required for the competence of several reproductive events [71]. Amino acids are the main nutritional elements for the oviduct and uterine histotroph and are an essential component in the amniotic and allantoic fluids, supporting the important role of amino acids for normal embryonic and fetal development. In cows, the concentration of methionine, histidine, and lysine in the uterine lumen has been found to increase more than 10-fold during embryo elongation (days 14 to 18). An inadequate supply of these amino acids can hinder the rapid growth of the embryo between days 14 and 19 in the pregnant cow and, afterward, the subsequent growth of embryonic, fetal, and placental tissues [72]. Feeding rumen-protected methionine is able to improve the embryonic size and pregnancy maintenance in multiparous cows [73]. In context, some fatty acids, specifically long-chain polyunsaturated fatty acids originated from oily seeds (linseed, soybean, rapeseed, and sunflower seed), fish oil, and fat-based feed additives (tallow and protected fats) can affect metabolism, hormonal balance, gonads functions, quality of gametes, embryo survival, and establishment of pregnancy in different farm animals [12,74,75,76]. Cows supplemented with linseed oil (source of linolenic, C18:3) tended (*p* = 0.07) to show higher plasma progesterone levels, and a higher conception rate on the first artificial insemination when compared with soybean supplemented cows and control cows [76]. Furthermore, some minerals, such as selenium, zinc, calcium, and phosphorus, have specific effects on reproduction in both males [77] and females [75]. As an example, feeding organic seleniumimprovesneutrophilfunction around parturition, immune responsivenessin multiparous cows, uterine health, and increasessecond-service pregnancy per artificial insemination [75].

Imbalanced nutrition causes body weight loss, poor body condition, delayed puberty, long days open, ovarian dysfunction, hormonal imbalances, and, thus, causes infertility. The adjustment of nutritional requirements of farm animals for energy and different nutrients (protein, fat, vitamins, and micro- and/or macro-minerals) is crucially required to achieve optimal reproductive performance [65]. Actually, meeting animals’ requirements during different reproductive windows is not always an easy matter and is greatly challenged by many limiting factors. In many cases, animals cannot obtain their optimal nutritional requirements and show symptoms of nutrient deficiency; however, the deficiency may not be due to the lack of offered feed and/or nutrients. In fact, nutrient utilization efficiency depends on many factors, such as feed intake, digestion and absorption efficiency, and the physiological and metabolic status of animals [65,66]. In most farm animals, the periods around mating, late pregnancy, and early lactation are associated with behavioral, metabolic, and physiological changes that may affect both feed intake and nutrients utilization [76]. For example, late pregnant animals, specifically ruminants, cannot obtain enough energy intake due to the limited capacity of rumen/stomach with pregnancy advancement, leading to a symptom of negative energy balance [78]. In such a state, providing high corn/grain-based diets results in several health problems and reproductive disorders, mainly aci0dosis. The symptoms of negative energy balance have been solved by increasing diet energy density by including fats, oils, glycerol, and propylene glycol [79]. However, improper use of such supplementations may drive negative effects on rumen microorganism action and rumen fermentation, impairing the overall performance of the animal [80]. For instance, glycerol is rapidly fermented in the rumen and negatively affects microbial fermentation [80]; furthermore, long-chain polyunsaturated fatty acids have toxic effects on rumen microorganisms too [81]. Therefore, the amounts of these supplementations should be restricted in the diet.Similarly, feeding diets high in crude protein or inadequate in fermentable carbohydratescan result in inefficient protein utilization and excess absorption of rumen ammonia [82]. Increased circulating ammonia levels can change the uterine environment and have toxic effects on an embryo, increasing the risks of embryonic mortality [83]. On the other hand, the biological role of some specific nutrients, such as unsaturated fatty acids and amino acids, could be impaired by the action of enzymatic hydrolysis of microorganisms. Rumen bacteria biohydrogenation toxic dietary unsaturated fatty acids to a saturated fatty acid to protect their cellular construction [81]. This mechanism results in a higher outflow of saturated fatty acids to the small intestine for digestion and absorption [84], decreasing the availability of these functional fatty acids to reproductive organs.

### 4.2. Nanotechnology Approaches for Improving Nutritional Management Outputs

Specific engineered nanomaterials can be implemented to provide various solutions to nutritional manipulation challenges raised during feeding producing animals (Table 2). In the field of animal nutrition, nano-encapsulation of active feed ingredients and processing of nano-minerals, in particular trace minerals of poor bioavailability, are rapidly growing technologies that are used to protect targeted feed ingredients from degradation (during processing, storage, and in the rumen) and to ensure effective and sustained delivery of valuable feed ingredients to target sites [85]. In addition, feed ingredients in nano-forms show novel physicochemical properties (mainly small size and high surface area) that improve its absorption, bioavailability, and utilization by animal tissues. However, up to date, studies showing the effect of nano-feed ingredients on animal reproduction are limited. Few studies have shown the positive effects of nano-minerals on the reproductive performance of farm animals [86,87,88,89].

In a study by El-Sherbiny et al. [81], nano-emulsification of soybean and fish oil, aimed at post-rumen delivery of long-chain polyunsaturated fatty acids, significantly increased the proportions of long-chain polyunsaturated fatty acids (oleic, linoleic, and linolenic acids) in the rumen fluid without negative effects on rumen fermentation efficiency. Similarly, Albuquerque et al. [90] used solid lipid nanoparticles (composed of arachidonic or stearic acids) to protect lysine from enzymatic hydrolysis by ruminal microbiota and to increase rumen-bypass of lysine to the small intestine. In this study, the lipid–lysine nano-formula showed stability against microbial hydrolysis in the rumen for up to 24h. In addition, Gawad and Fellner [85] applied encapsulation techniques to protect glycerol from microbial fermentation using alginate or alginate–chitosan; results showed the efficiency of alginate–chitosan mixture to minimize encapsulated glycerol release into the rumen culture and to increase amounts of glycerol delivered to the lower digestive tract; however, the particle size was not at nano-scale [85]. Having in mind these findings, nanotechnology can improve nutrient utilization and the bioavailability of specific nutrients needed during specific periods of the reproductive cycle, by solving the imbalance between elevated needs of energy and active feed ingredients (amino acids, fatty acids, and minerals), and other biological processes, such as rumen fermentation.

In addition, the opportunity to use lowerquantitiesof nano-feed ingredients to get the same effects as ordinary forms may decrease feeding costs and the release of some materials, mainly minerals, to the environment decreasing pollution.

## 5. Management of Reproductive-Related Diseases

### 5.1. Importance and Challenges of Antibiotic Applications

Animal production, and specifically reproduction, is usually associated with the episode of reproductive diseases. Postpartum diseases, specifically endometritis caused by different bacterial species (mainly *Escherichia coli*, *Staphylococcus aureus*, *Bacillus cereus*, *Pseudomonas aeruginosa*, *Prevotella melaninogenica*, and *Arcanobacterium pyogenes*), are accompanied by impaired reproductive performance reflected in reduced conception rate and increased risk of reproductive culling [91]. The hazard of pregnancy, days elapsed from calving until pregnancy, reached 0.60, 0.31, and 0.24 in cows with metritis, clinical end metritis, and subclinical end metritis, respectively, when compared to healthy herd mates [92]. Pregnancy-associated diseases, such as toxoplasmosis (*Toxoplasma gondii*) and neosporosis *(Neospora caninum)*, are protozoan diseases that lead to significant economic loss in farm ruminants. Worldwide, toxoplasmosis infection is a zoonosis that mainly causes reproductive failure in small ruminants, whereas neosporosis infection is a common zoonosis that causes abortion in cattle [93]. In dairy farms, bovine mastitis, mainly caused by *Staphylococcus aureus* causes significant economic losses due to severe declines in milk yield (about 380 tons of milk are lost every year in the world), dumped milk, reproductive disorders, and expenses paid to the replacement of infected animals, increased costs of pharmacologic costs, and replacing tainted milk [15,94]. Additionally, the contamination of raw milk with *Staphylococcus aureus* raises public health problems throughout the food chain.

Overall, these diseases have negative impacts on the animals’ reproductive efficiency and welfare, public health, and the final profit of the production process. The clinical symptoms of most of these bacterial and/or zoonotic diseases are mediated directly by microbial products (endotoxin) and tissue damage, or indirectly by inflammatory (cytokines and eicosanoids) and/or oxidative stress (nitric oxide) mediators [93]. These changes have a negative impact on sperm function and quality (sperm motility and sperm phagocytosis), ovarian function (follicular steroidogenesis and growth, ovulation, and ova competence development to blastocyst), uterine competence (implantation failure), and embryonic development (retarded development into blastocyst) [15,92].

Currently, antibiotic-based therapy is the commonly recommended therapy for tackling different microbial/protozoan diseases, including reproductive-related diseases. The effectiveness of antibiotics-based therapies is controlled by the pharmacokinetics of the drug. The delivery of antibiotics into targeted infected sites depends on the rate of absorption and distribution of the drug, which can be limited by different biological factors as the stability of the antibiotics against degradation by gastrointestinal enzymes (oral administration), blood hydrolytic enzymes (parenteral administration), drug solubility, and thus cellular uptake and bioavailability. Furthermore, some diseases cause fibrous damage in infected tissues limiting the penetration of the antibiotics into infected sites when local treatment is applied, such as in the direct infusion of the drug into the uterus in endometritis cases or through teats in mastitis cases [15,95].

Despite the limiting biological and therapeutic efficiency of the current antibiotics-based therapies, emerging concerns regarding the development of antimicrobial-resistant species add other limitations on the applications of antibiotic-based therapies, specifically in food-producing farm animals. The fear of developing more wild pathogenic microbial species, creating infectious and cross-transited microbial species, transferring of antibiotic residues into animal products (meat and milk), and the release of antibiotics into the environment are all aspects that should be taken into account [96]. Antimicrobial resistance leads not only to an encumbrance on public health but also extends to the risk of therapy failure and repeated infection, along with subsequent economic impacts. Actually, these factors make the treatment of reproductive-related diseases (mastitis [15], toxoplasmosis, and neosporosis [93]) by antibiotics a controversial strategy. Accordingly, novel, safe, and effective antibiotics-based therapeutic approaches are needed, particularly when treatments are directed to food-producing farm animals [93].

### 5.2. Nanotechnology Approaches

Many studies have shown the opportunity of using many engineered nanomaterials (e.g., liposomes, polymeric nanoparticles, solid lipid nanoparticles, nanogels, and inorganic nanoparticles), which are synthesized with specific physicochemical properties to overcome the therapeutic limitations of antibiotics-based therapies [94,97,98,99].

The use of nano-formula for antibiotics-based therapies may offer additional advantages over conventional antibiotics formula, such as (1) reducing the dose of the antibiotic, (2) allowing efficient delivery of the antibiotic to the infected sites, (3) shortening the therapeutic timing and side effects, and (4) preventing burst release and degradation of the antibiotic [94]. Nanomaterials may be protective against the rapid degradation of the antibiotic and may improve its delivery to the infected site, but, moreover, nanomaterials themselves could be engineered to show cytotoxic and destructive properties against microorganisms. Moreover, some nanoparticles have destructive effects on the bacterial cell membrane, enzymes, and functional and structural cell proteins mainly through evoking cellular oxidative pathways, in addition to their ability to inhibit the formation of bacterial biofilm, to induce changes in the gene expression, and to stimulate innate and adaptive immunity [97]. Additionally, nanoparticles could be engineered to hinder the bacterial adhesion, colonization, and biofilm development of bacteria [15]. Furthermore, nanomaterials have the ability to incorporate one or more drugs without any effect on the structure of the compound but increasing its pharmacological action [98].

Specifically, in swine, enrofloxacin antibiotic is used to treat several bacterial infections, such as *Pasteurella*, *Mycoplasma*, *Escherichia coli*, or *Salmonella*, with an intramuscularly recommended dose average between 2.5 to 5 mg enrofloxacin/kg Bw/day for 3 to 5 days. Paudel et al. [99] showed that enrofloxacin-loaded poly(lactic-co-glycolic acid) nanoparticles may be delivered orally in a suspension in drinking water, and the minimum inhibitory concentration against *Escherichia coli* was reduced by 23% compared to free enrofloxacin alone. Such finding, combined with increased bioavailability, maybe an interesting first step to reduce the dose of enrofloxacin and, therefore, its side effects (including the propagation of antibiotics resistance). El-Zawawy et al. [100] reported that incorporating triclosan into the lipid bilayer of liposomes allowed its usein lower doses, which in turn reduced its biochemical adverse effects. In another study, sodium dodecyl sulfate-coated atovaquone nanosuspensionsconsiderably increased the therapeutic efficiency against experimentally acquired and reactivated toxoplasmosis by improving the passage of gastrointestinal and blood–brain barriers [101]. Similarly, tilmicosin (a semi-synthetic macrolide antibiotic)-loaded hydrogenated castor oil with lower dosage showed better therapeutic efficacy than free tilmicosin for *Staphylococcus aureus* mastitis infectiondue to the enhanced bioavailability and sustained-release performance [102]. Recently, nano drugs have also been used as astrategy to solve the multi-drug resistance and intracellular persistence of *Staphylococcus aureus*, which is associated with the subclinical and relapsing infection of bovine mastitis [94]. Yang et al. [13] showed the possibility of prolonging post-antibiotic effects and thus dosing intervals when amoxicillinnanoparticles are used for treating bovine mastitis. This would decrease the rate of antibiotic use and the costs of medication.

Recently, the combination of the advantages of nano-drug delivery technology and alternative medicine, which depends on the usage of natural products with antimicrobial activity, opens the way to innovative natural and safe antibiotic alternatives. Numerous studies have shown probiotic species, microbial extracts, and plant secondary metabolites (essential oils and polyphenols) as potential antimicrobial agents [103,104]. A nano-formulacontaining 0.4–10% of oregano oil was developed for treating dairy cow endometritis. The uterine infusion of this nano-formula for 2–5 days showed a remarkable curative effect, being able to diminish inflammation, sterilizing uterus, draining pus by contracting the uterus, and promoting the recovery of the uterine function (Patent: CN104288222A, china https://patents.google.com/patent/CN104288222A/en; accessed on 12 February 2021). In another study, poly(lactic-coglycolic) acid(PLGA)-epigallocatechin gallate-deoxycyclin nanoparticles have been successfully used as an assisted-endometritis therapy [105]. In context, chitosan-TPP nanoparticles have been used for treating mastitis [106].

Additionally, many metal nanoparticles, such as silver oxide (Ag_2_O), gold (Au), zinc oxide (ZnO), titanium dioxide (TiO_2_), and copper oxide (CuO), have shown effective antimicrobial activity against a broad spectrum of microorganisms [19]. These approaches may give an opportunity to completely substitute antibiotics-based treatments with more safe therapies. The emergence of biological biosynthesis procedures of nanometal (silver) using microorganisms (*Escherichia coli*, *Acinetobacter species*, *Staphylococcus aureus*, *Pseudomonas aeruginosa*, and *Klebsiella pneumoniae*) and/or natural reducing agents (polyphenols, flavonoids, and phenolic biomolecules of Camellia, green tea, and black tea leaf extracts)has encouraged the use of nano metals as an alternative to antibiotics, meeting both therapeutical and environmental aspects [103,107]. In this context, apigenin (a polyphenolic compound) was successfully used to synthesis silver nanoparticles with a size of 10 nm.These nanoparticles showed antimicrobial activity against pathogenic bacteria *Prevotella melaninogenica* and *Arcanobacterium pyogenes* isolated from endometritis infected uterine discharges by inhibiting cell viability and biofilm formation in a dose-and time-dependent manner [91]. Similarly, Yuan et al. [108] confirmed the antibacterial activity of biologically synthesized silver nanoparticles against two multiple drug-resistant strains of *Pseudomona saeruginosa* and *Staphylococcus aureus* isolated from mastitis-infected goats milk samples.

Regarding the toxicity of such nanomedicines to animal tissues, Radzikowski et al. [109] confirmed the potential of commercially available silver nanoparticles, copper nanoparticles, and their combination in decreasing the viability of mastitis-borne pathogens without showing toxic effects on mammary gland tissues. Furthermore, Paudel et al. [99] confirmed the lower toxicity of enrofloxacin entrapped nanoparticles to mammalian cells relative to a free drug as the incorporation of the drug into the PLGA matrix minimized the production of reactive oxygen speciesevoked by the antibiotic.

A summary of studies on the nano drugs developed to treat reproductive-related diseases is shown in Table 3.

## 6. Conclusions

In this review, we illustrated the existing challenges and limitations of the most commonly applied reproductive management strategies in farm animals. As illustrated, the efficiency of the reproductive management strategies is restricted by the ease of application in the field scale, physiological status and behavior of animals, drug availability and uptake, in addition to environmental aspects, such as the release of antibiotic/hormone residues. Nanotechnology presents innovative and alternative solutions, such as that posed for solving challenges of the male effect by using an aerosol nano-drug delivery system of pheromones. Applying nanotechnology may also solve the problem of the lack of some hormones in the near future by changing the pharmacokinetics and pharmacodynamicsbehavior of hormones allowing the use of the hormones in other unconventional biological applications. Furthermore, challenges related to nutritional management can also be solved mainly through improving nutrients bioavailability during sensitive reproductive windows, which may interfere with nutrients availability and utilization. Lastly, nanotechnology can improve the efficiency of the antibiotic and/or creating natural antibiotic alternatives that may restrict the development of antibiotics resistant microbial species.However, this review presents ambitious solutions for the existing reproductive management challenges in the light of available literature; more studies are required to test the efficiency of such strategies.

## Figures and Tables

**Table 1:** Characteristics and expected advances by the implementation of nanotechnologies in the use of reproductive hormones for farm animals’ reproductive management.

Figure	Technique	Particle Characteristics	Expected Advances
GnRH-chitosan-TPP NPs [7]	Ionic-gelation	Size = 212 nm, PdI = 0.295, Zp = 8.0 mV, EE = 90%	⮚ Optimizing route of administration ⮚ Decreasing dosage ⮚ Increasing bioavailability ⮚ Increasing animal welfare
GnRH-chitosan-TPP NPs [53]		Size = 93.91 nm, PdI = 0.302, Zp = 11.6mV, EE = 91.2%	
GnRH-chitosan-dextran sulfate NPs [51]	Ionic-gelation	EE = 40−50%	
hCG-chitosan-TPP NPs [35]	Ionic-gelation	-	
P_4_-chitosan-TPP-Tween 80 [61]	Spray-drying	Size = 1 and 7 μm, EE = 69–75%	⮚ Improving environmental and human health
P_4_-polymethyl-methacrylate-nanospheres [57]	Miniemulsion polymerization	size = 150–200 nm, EE > 69%	
P_4_-polymethyl-methacrylate-nanocapsules [57]		size = 240–300 nm, EE > 90%	
P_4_-polylactic acid NPs [55]	Solution blow spinning	Size = 289–441 nm	
Melatonin-loaded lipid-core Nps [58]	Interfacial deposition of polymer	size = 168 nm PdI = 0.08	⮚ Enhancing quality and increasing production rates of bovine blastocysts produced by in vitro fertilization
Melatonin loaded-lipid (olive oil) NPs [60]	Hot homogenization-ultrasonication	Size = 119nm, PdI = 0.09, EE = 94%	⮚ Sustained release during IVF

GnRH = gonadotropin releasing hormone, TPP = tripolyphosphate, NPs = nanoparticles, hCG = human chorionic gonadotropin, P_4_ = progesterone, PdI = polydispersity, Zp = zeta potential, and EE = encapsulation efficiency, and IVF = in vitro fertilization.

**Table 2:** Characteristics and expected advances by the implementation of nanotechnologies in nutritional management for farm animals’ reproductive management.

Formula	Technique	Particle Characteristics	Expected Advances
Zinc oxide NPs [89]	Commercial product	Size = 30.92 nm Zp = 32.16 mV	⮚ Increase bioavailability and reduce the negative effects of toxic concentrations in in vitro reproductive assisted techniques
Selenium oxide NPs [89]	Commercial product	Size = 78.47 nm Zp =−20.36 mV	
Selenium oxide NPs [86]	Chemical reduction method using ascorbic acid and acacia gum	Size = 45.00 nm	⮚ Increase absorption and bioavailability and reduce the negative effects of toxic concentrations during late pregnancy
Fish oil or soy oil -in-water NPs Soy oil-fish oil or rapeseed-fish oil-in-water NPs [81]	Nanoemulsion	-	⮚ Improve post-ruminal supply of PUFA ⮚ Decreased transformation rate of PUFA to SFA in the bio-hydrogenation environment
Solid lipid-lysine NPs [90]	Ultrasonic processor	Size = 200–500 nm Zp = < −30 mV EE = 40−90%	⮚ Improve post-ruminal supply of lysine amino acid
Alginate-chitosan-glycerol NPs [85]	Ionic-gelation	Size = 3 mm EE = 78.1%	⮚ Encapsulating glycerol to bypass rumen fermentation

Nps = nanoparticles, Zp = zeta potential, EE = encapsulation efficiency, PUFA = poly unsaturated fatty acids, and SFA = saturated fatty acids.

**Table 3:** Summary of studies on the nano drugs developed to treat reproductive-related diseases.

Type of Drug	Formula	Technique	Particle Characteristics	Drug Activity	Usage
Antibiotic [99]	Enrofloxacin- poly lactic-co-glycolic acid NPs	-	Size = 102 nm PdI = 0.095 Zp = −32 mV	Antimicrobial agent against *Staphylococcus aureus, Escherichia coli*	Endometritis and mastitis treatment
Antibiotic [102]	Tilmicosin-loaded hydrogenated castor oil NPs	Hot homogenization and ultrasonication	Size = 343 nm PdI = 0.33 Zp = 7.9 mV EE = 60.4%	Antimicrobial agent against *Staphylococcus aureus*	Mastitis treatment
Antibiotic [100]	Triclosan-loaded liposome NPs	Dehydration-rehydration	Size = 53.3 nm EE = 90%	Antimicrobial agent against *Toxoplasma gondii*	Toxoplasmosis treatment
Antibiotic [101]	Atovaquone-poloxamer 188 - sodium dodecyl sulfate	-	-	Antimicrobial agent against *Toxoplasma gondii*	Toxoplasmosis treatment
Nitric oxide (NO) [110]	NO-alginate-chitosan NO-chitosan-TPP	-	Size= 270–375 nm PdI=0.27–0.31 Zp = 16−17 mV	Antimicrobial agent against *Staphylococcus aureus, Escherichia coli*	Mastitis treatment
Metal [91]	Silver NPs	Biosynthesis by apigenin	Size = 10 nm	Antimicrobial agent against *Prevotella melaninogenica* and *Arcanobacterium pyogenes*	Antibiotic alternative for endometritis treatment
Metal [108]	Silver NPs	Biosynthesis by quercetin	Size = 20 nm Zp= 37.7mV	Antimicrobial agent against *Staphylococcus aureus* and *Pseudomonas aeruginosa*	Antibiotic alternative for mastitis treatment
Chitosan [106]	Chitosan-TPP Nps	Ionotropic gelation	Size = 19.1 nm PdI = 0.41 Zp = 49.9 mV Yield particle = 92.8%	Antimicrobial agent against *Pseudomona* sp.	Antibiotic alternative for mastitis treatment
Antibiotic + polyphenol [105]	Poly(lactic-co-glycolic) acid-epigallocatechin gallate- doxycycline Nps Singh et al., 2015	Modified double emulsion solvent evaporation/extraction technique	Size = 176 to 211 nm PdI = 0.124 to 0.466 EE= 78.5 to 86.3%	Anti-inflammatory agent	Assisted-endometritis therapy
Essential oil ^1^	Oregano oil Nps	-	-	Antimicrobial agent against *Staphylococcus aureus, Escherichia coli, Streptococcus* spp.	Antibiotic alternative for endometritis treatment

Nps = nanoparticles, Zp = zeta potential, PdI = Polydispersityindex, and EE = encapsulation efficiency.^1^ https://patents.google.com/patent/CN104288222A/en; accessed on 12 February 2021.
